# Publisher Correction: Novel interferon-sensitive genes unveiled by correlation-driven gene selection and systems biology

**DOI:** 10.1038/s41598-021-99452-0

**Published:** 2021-09-30

**Authors:** Cristina Cheroni, Lara Manganaro, Lorena Donnici, Valeria Bevilacqua, Raoul J. P. Bonnal, Riccardo L. Rossi, Raffaele De Francesco

**Affiliations:** 1 Virology, Istituto Nazionale Genetica Molecolare “Romeo ed Enrica Invernizzi”, 20122 Milan, Italy; 2 Integrative Biology, Istituto Nazionale Genetica Molecolare “Romeo ed Enrica Invernizzi”, 20122 Milan, Italy; 3 Bioinformatics, Istituto Nazionale Genetica Molecolare “Romeo ed Enrica Invernizzi”, 20122 Milan, Italy; 4 Department of Pharmacological and Biomolecular Sciences (DiSFeB), University of Milan, Milan, Italy; 5 Present Address: High Definition Disease Modelling Lab, Stem Cell and Organoid Epigenetics, IEO, European Institute of Oncology, IRCCS, Milan, Italy; 6 Present Address: Department of Oncology and Hemato-Oncology, University of Milan, Milan, Italy; 7 Present Address: FIRC Institute of Molecular Oncology (IFOM), 20139 Milan, Italy

Correction to: *Scientific Reports* 10.1038/s41598-021-97258-8, published online 10 September 2021

In the original version of this Article, Affiliation 5 and 6 were incorrectly given as ‘Present address: High Definition Disease Modelling Lab, Stem Cell and Organoid Epigenetics, Department of Oncology and Hemato-Oncology, IEO, European Institute of Oncology, IRCCS, University of Milan, Milan, Italy’. The correct affiliations are listed below:

Present address: High Definition Disease Modelling Lab, Stem Cell and Organoid Epigenetics, IEO, European Institute of Oncology, IRCCS, Milan, Italy.

Present address: Department of Oncology and Hemato-Oncology, University of Milan, Milan, Italy.

In addition, Raoul J. P. Bonnal was incorrectly affiliated with ‘Present address: High Definition Disease Modelling Lab, Stem Cell and Organoid Epigenetics, Department of Oncology and Hemato-Oncology, IEO, European Institute of Oncology, IRCCS, University of Milan, Milan, Italy’. The correct affiliations are listed below.

Integrative Biology, Istituto Nazionale Genetica Molecolare “Romeo ed Enrica Invernizzi”, 20122, Milan, Italy.

Present address: FIRC Institute of Molecular Oncology (IFOM), 20139, Milan, Italy.

The original Article has been corrected.

